# A database of animal metagenomes

**DOI:** 10.1038/s41597-022-01444-w

**Published:** 2022-06-16

**Authors:** Ruirui Hu, Rui Yao, Lei Li, Yueren Xu, Bingbing Lei, Guohao Tang, Haowei Liang, Yunjiao Lei, Cunyuan Li, Xiaoyue Li, Kaiping Liu, Limin Wang, Yunfeng Zhang, Yue Wang, Yuying Cui, Jihong Dai, Wei Ni, Ping Zhou, Baohua Yu, Shengwei Hu

**Affiliations:** 1 State Key Laboratory of Sheep Genetic Improvement and Healthy Production, Xinjiang Academy of Agricultural and Reclamation Science, Shihezi, Xinjiang China; 2 College of Life Sciences, Shihezi University, Shihezi, Xinjiang 832003 China; 3 College of Information Science and Technology, Shihezi University, Shihezi, Xinjiang 832003 China; 4 College of Animal Science and Technology, Shihezi University, Shihezi, Xinjiang 832003 China

**Keywords:** Databases, Data integration

## Abstract

With the rapid development of high-throughput sequencing technology, the amount of metagenomic data (including both 16S and whole-genome sequencing data) in public repositories is increasing exponentially. However, owing to the large and decentralized nature of the data, it is still difficult for users to mine, compare, and analyze the data. The animal metagenome database (AnimalMetagenome DB) integrates metagenomic sequencing data with host information, making it easier for users to find data of interest. The AnimalMetagenome DB is designed to contain all public metagenomic data from animals, and the data are divided into domestic and wild animal categories. Users can browse, search, and download animal metagenomic data of interest based on different attributes of the metadata such as animal species, sample site, study purpose, and DNA extraction method. The AnimalMetagenome DB version 1.0 includes metadata for 82,097 metagenomes from 4 domestic animals (pigs, bovines, horses, and sheep) and 540 wild animals. These metagenomes cover 15 years of experiments, 73 countries, 1,044 studies, 63,214 amplicon sequencing data, and 10,672 whole genome sequencing data. All data in the database are hosted and available in figshare 10.6084/m9.figshare.19728619.

## Abstract (summary)

**Table:** 

Measurement(s)	Metagenome metadata
Technology Type(s)	Collection and integration the metagenomic information of multiple animal species
Factor Type(s)	animal
Sample Characteristic - Organism	animal
Sample Characteristic - Environment	metagenome
Sample Characteristic - Location	United States of America • People’s Republic of China • Canada

## Background & Summary

Microorganisms play essential roles in specialized niches, including internal host biology as well as the external environment. Microbes are found in diverse habitats, including deep seas, saline marshes, and glaciers^1^. The roles of microorganisms in biodiversity have become a focus of interest for researchers due to their omnipresence^2^. This diversity represents a vast genetic resource that could be exploited for the discovery of novel genes, biomolecules for metabolic pathways, and potentially valuable end-products^3^. The structure and function of the microbial community has received significant attention for decades, notably in association with research concerning human microbiota^4^. However, veterinarians, animal nutritionists, and microbiologists have begun to focus on studying the microbes of domestic (horses, pigs, and ruminants) and wild animals^5^. For domestic animals, a better understanding of disease-causing microbes of livestock can contribute to achieving the goals of better foods and a cleaner environment^6^. This is not only conducive to the healthy development of domestic animal and poultry breeding industries but also reduces the risk of food-borne diseases being transmitted to humans, which is conducive to public health security^7^. Regarding wild animals, wildlife microbiota are also natural hosts for animal and human pathogens; mapping their distributions can shed light on the timing and pathways of their transmission to humans, as is the case in the current COVID-19 pandemic^8^.

With the development of ultra-high throughput metagenomic sequencing technologies, including 16s rRNA gene sequencing and whole-genome sequencing, the number and scope of metagenomic sequencing projects have increased rapidly^9^. This has led to an exponential growth of metagenomics data under different experimental conditions. Therefore, metagenomic data contain an overwhelming volume of complex information, posing challenges not only for data storage but also for metadata annotation and management. Several pioneering studies have been designed to construct resources for storing raw sequencing data, including the National Center for Biotechnology Information (NCBI)^10^, the Sequence Read Archive (SRA)^11^, and the European Nucleotide Archive (ENA)^12^. These public resources contain human and animal metagenomic information that will serve as an important reference for current studies^13^. For instance, a previous study^14^ meta-analyzed 20 publicly available datasets from 16S rRNA gene-sequencing studies of the swine gut microbiota and demonstrated that GI tract location was the strongest predictor of the swine gut microbiota composition. In addition, eight genus-level gut microbes were identified that could serve as potential markers of swine gut microbiota. Therefore, the inclusion of comprehensive metadata accompanying the sequencing dataset will enhance future meta-analyses and allow researchers to directly compare their results with those of similar studies. Although some databases provide all raw data from the relevant experiments, it is still difficult for users to compare, classify, analyze, and store the data, given the limitations of computing resources and devices^15^. To address this problem, various databases have been created. For example, the HumanMetagenome DB 1.0 simplifies the identification and use of public human metagenomes^16^. Studies such as TerrestrialMetagenome DB^17^ and PlanetMicrobe^18^ have been reported. However, there are currently few reports concerning research datasets of animal metagenomes.

The present study found that large-scale population research can usually capture the orientation law of organisms to a certain extent^19–21^; for example, human microbiome composition^22–24^. The scale of the research also means that multiple effects can be compared across the same set of samples; for example, rearrangement of the gut bacterial ecosystem during the weaning transition in pigs^25^. Nevertheless, large-scale data integration can provide a common framework for comparison effects. The establishment of the animal metagenomic database aims to provide large-scale data integration related to the animal microbial datasets^26^. The goal is to provide researchers with a browsing interface and multiple consideration options so that users can quickly obtain the information they seek. For example, considering large-scale population data is better suited to achieving common research goals and to understanding the factors that are related to the changes of animal intestinal microbial flora^27^.

Here, we focused on collecting and integrating the metagenome content of multiple animal species to help users understand the ecological underpinnings of microbiomes. The main merit of this work lies in the integrated implementation of the basic project information in the form of a very robust and user-friendly interface^28^. To summarize, we have organized and integrated the information in the data for ease of use by matching experimental items to microbial enrichment variation; browsing and search functions were implemented, making the database easily used by researcher and biologists. In the future, we will continue to improve the animal metagenomics database, adding common domestic animals in terms of species, and we plan to update the database every two years.

## Methods

### Database construction

**Fig. 1:**
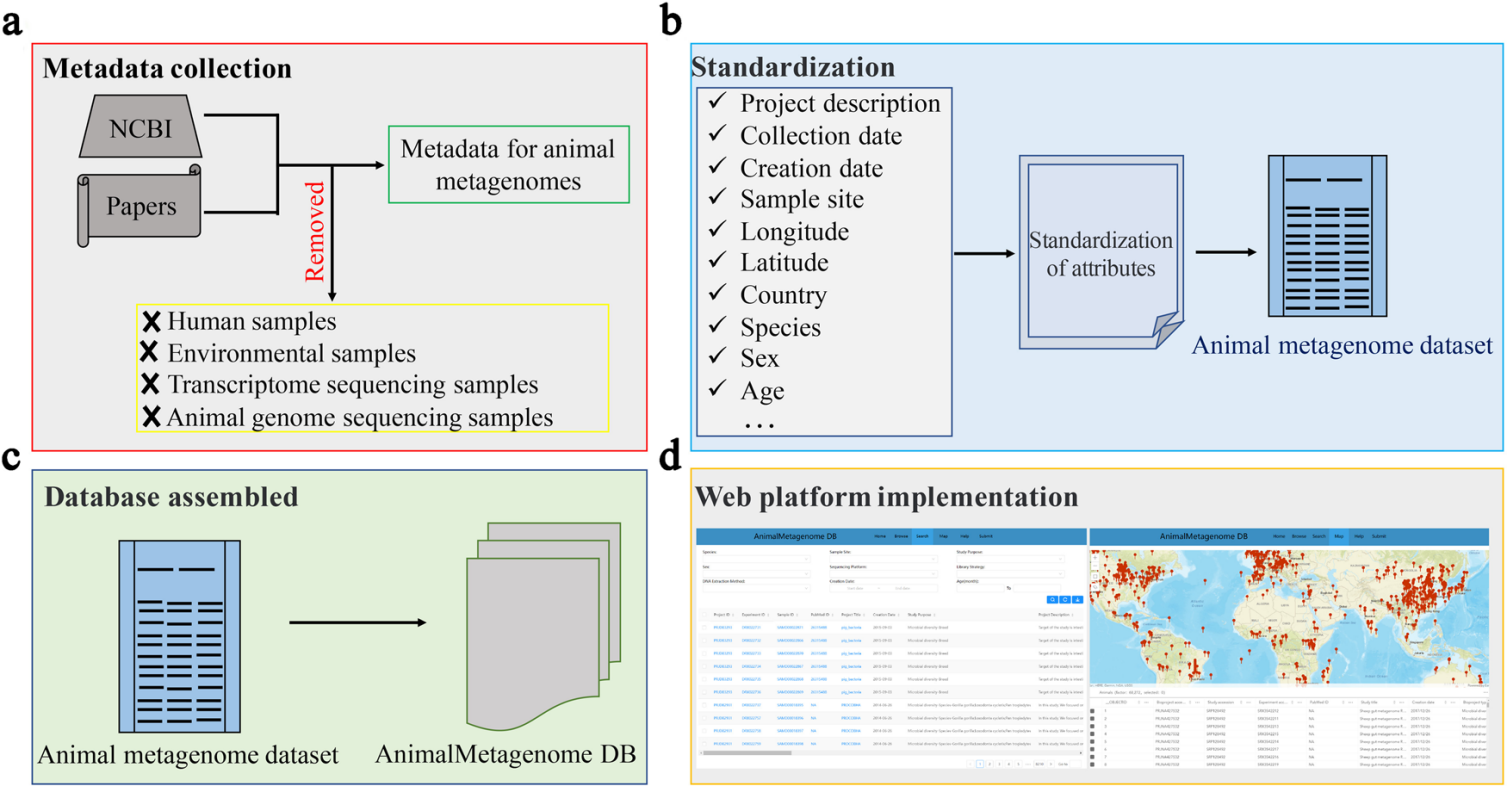
Overview of the AnimalMetagenome DB construction method. (**a**) Metadata collection for animal metagenomes. (**b**) Standardization of attributes. (**c**) Database platform construction. (**d**) The Animal Metagenome DB web implementation.

The construction scheme of the AnimalMetagenome DB is shown in Fig. 1. First, we collected the metadata of animal metagenomes from the NCBI database and from published articles. Subsequently, we removed human samples and environmental samples as well as non-metagenomic datasets. Then, we collected project information and standardized the sample attributes. Finally, all data were assembled into the database system, and the web platform was implemented.

### Data collection

All data contained in the AnimalMetagenome DB were collected manually. First, we searched for projects related to four domestic animals: pigs, horse, bovines, and sheep, and wild animals from the NCBI BioProject database (https://www.ncbi.nlm.nih.gov/bioproject) using the keywords “Pig”, “Swine”, “Bovine”, “Cattle”, “Horse”, “Sheep”, and “Wild animal”. Then, we screened out projects related to animal metagenome research and collected information about these projects, including project description, project accession number, project creation date, and the number of samples contained in the project. Second, according to the project accession number, we collected data of all samples of this project from the NCBI BioSample database (https://www.ncbi.nlm.nih.gov/biosample) and collected other available data of these samples from published articles, such as PubMed ID, DNA extraction method, and the geographic location where the sample was collected. Then, we checked the information collected from the NCBI BioSample database with that of the original article to ensure the accuracy of the data. In addition, we integrated all information about the Experiments and Runs associated with the samples from the NCBI SRA database (https://www.ncbi.nlm.nih.gov/sra/) and matched the sample information with the information of Experiment and Run according to the sample accession number. Finally, the metadata of all animal metagenomes were counted to indicate that the collection was complete.

### Confirmation of animal metagenomes based on the metadata

We confirmed animal metagenomic samples by checking the information contained in the experiment title, sample title, and sample name. After screening, we deleted environmental samples, human samples, animal genome sequencing samples, and transcriptome sequencing samples in the animal metagenomic projects.

### Standardization of attributes

In the BioSample database, the sample attributes are not standardized, and the attribute names are displayed in many different ways. Therefore, to unify the description of the sample attributes, we referred to the curatedMetagenomicData^29^ to standardize 10 different attributes, including species, sample site, sex, age, host phenotype, experimental condition, sample collection date, sample geographic location, longitude, and latitude. For species, we divided domestic animals into four types: pigs, bovines, horses, and sheep, and referred to the NCBI Taxonomy database (https://www.ncbi.nlm.nih.gov/taxonomy) to classify wild animals by class, order, family, genus, and species. The sample site1 (host body site) was divided into five main categories: gut (e.g. cecum, ileum), stomach (e.g. abomasum, rumen), skin, oral cavity, and bio-fluids (e.g. saliva, milk). We refer to HumanMetagenomeDB^16^ to uniformly convert geographic locations to countries. Meanwhile, geographic location coordinates (longitude and latitude) were standardized to decimal format (rounded to two decimal places). The date was standardized in accordance with the international standard ISO 8601 (YYYY-MM-DD). The age of animals was converted to months, rather than days, weeks, or years. In addition, we referred to the Animal QTLdb database (https://www.animalgenome.org/QTLdb) to classify the study purpose of the project, which comprised health traits, production traits, microbial diversity, and life history traits. Health traits was divided into disease, immune capacity, and pathogens and parasites; life history traits included weaning; and production traits included growth, feed efficiency, feed intake, methane emission, metabolism, and meat quality.

### Web platform implementation

The AnimalMetagenome DB web architecture consisted of the front-end Vue and back-end SSM. Vue is a progressive framework for building user interfaces. It uses a data-driven approach that is different from the traditional dom-driven JavaScript. The back end was written in Java, and the integration framework of Spring + Spring MVC + Mybatis was chosen for development. The database was implemented in MySQL, a cross-platform, safe, and efficient database system.

## Data Records

Information about metadata in the AnimalMetagenome DB was manually collected from BioProject, BioSample^10^, SRA^11^, Taxonomy^30^, PubMed, and Google Scholar databases. Each entry contains four parts: sample information (sample ID, species, sample site, DNA extraction method, sex, age, collection date, geographic location, sample type), sequencing attributes (experiment ID, instrument, library source, library strategy, total size, total spots, total bases), project information (project ID, project title, project description, study purpose, creation date), and literature information (PubMed ID). Table 1 shows an overview of the attributes in the AnimalMetagenome DB.

**Table 1:** List of attributes present in the AnimalMetagenome DB.

Attributes	Definition
Project ID	Project ID from the NCBI BioProject database.
Study accession	Study ID from the NCBI SRA database.
Experiment ID	Metagenomic library ID from the NCBI SRA database.
PubMed ID	Article’s pubmed ID, if available.
Project title	Title of the project.
Creation date	Date when the project was created.
Project description	Project’s abstract.
Sample ID	Sample ID from the NCBI BioSample database.
Sample site	Origin of the sample based on the host body site.
Sex	Physical sex of the host.
Age	Age of the host at the time of sampling.
Collection date	Date of sample collection.
Condition	The information about the host’s experimental treatments.
Pheotype	Phenotype of the host.
Breed	Breed of animal.
Instrument	Sequencing platform.
Library strategy	Strategies for building metagenomic libraries.
Total size	Total number of reads present in the library.
Total bases	Total number of base pairs present in the library.
Species	Animal’s species name.
Geographic location	Location (country) where the sample was collected.
Latitude	Geographic coordinate of latitude in decimal degrees where the sample was collected.
Longitude	Geographic coordinate of longitude in decimal degrees where the sample was collected.
Study purpose	To classify the study purpose of the project based on the Animal QTLdb database. The classification of the bioproject type was made as to facilitate project clustering.

The current version of the data contains 5 Excel files, which are the metagenome metadata of pigs, horse, bovines, sheep and wild animals. These data are publicly available free-of-charge from the Figshare repository^31^. At the same time, these data can also be browsed, searched and downloaded through our online database website. AnimalMetagenome DB is freely available at http://animalmetagenome.com/.

## Technical Validation

### Contents of the database

**Fig. 2:**
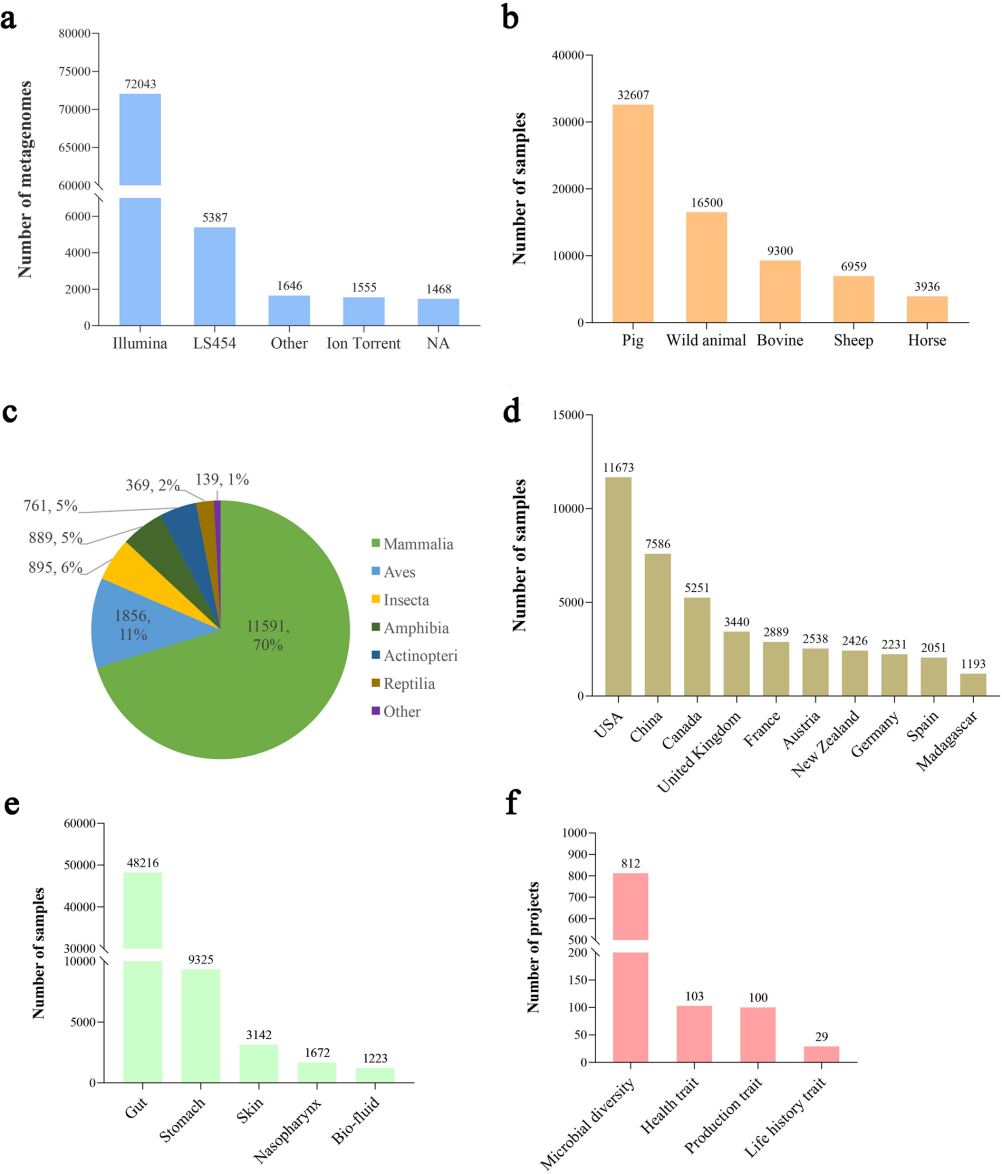
Selective statistics of the AnimalMetagenome DB content. (**a**) The distribution of sequencing platforms in the database. (**b**) The distribution of metagenome samples from different host sources. (**c**) The distribution of sample sites (top 5). (**d**) The distribution of wild animals at the class level. (**e**) The distribution of metagenome samples collected in different countries (top 10). (**f**) The distribution of different project research types.

The AnimalMetagenome DB version 1.0 includes metadata of 82,097 metagenomes from more than 540 species of animals, covering 15 years of experiments from May 2006 to May 2021. Of these metagenomes, 77% (63,214) were contained amplicon sequencing data, and 13% (10,672) were whole genome sequencing data. Furthermore, most libraries were sequenced using Illumina sequencing technology (88%), followed by LS454 sequencing technology (7%), Ion Torrent sequencing technology (2%), and other (2%) (Fig. 2a).

The database comprises 69,302 samples from 1,044 projects, of which 32,607 samples were from pigs; 16,500 samples were from wild animals; 9,300 samples were from bovines; 6,959 samples from sheep, and 3,936 samples were from horses (Fig. 2b). To facilitate the search by species, the wild animals were classified by class, order, family, genus, and species. At the level of class, the majority of the wild animal samples were from mammal species, followed by Aves species (Fig. 2c). The DNA extraction methods of some samples in the database were marked, and the DNA of most samples was extracted by DNA extraction kit (86.8%), followed by the phenol-chloroform method (5.5%), and the repeated bead beating plus column (RBB + C) method (3.9%). In addition, the host gender of some samples was also annotated, with 13.3% (9,348) of the samples derived from females and 12.7% (8,774) from males. According to the sample geographic location, metagenomes covered 73 countries; most of the samples were from the United States of America (USA), with 20.3% of the annotated samples, followed by the People’s Republic of China, with 13.2% of the annotated samples (Fig. 2d). According to the sample site information, 69.5% of the samples were from the gut, followed by the stomach at 13.5% (Fig. 2e). From the perspective of different study methods, the database also contains information about the host’s experimental treatments (for example, different diet types: “high-fiber diet” and “low-fiber diet”), and information on chemical administration (for example, the host used antibiotics, drugs, or other specific chemicals).

According to purpose of projects, the 1,044 projects contained in the database were classified into four main types: microbial diversity, production traits, health traits, and life history traits (Fig. 2f). For the health traits, 26 different diseases were included (for example: “diarrhea”, “flu”), of which diarrhea-related projects were the most numerous. Among projects on production traits, 42% of the studies were related to metabolism, followed by the studies on methane emission accounting for 20%, feed efficiency accounting for 20%, and feed intake accounting for 6%.

### Functions of the database

**Fig. 3:**
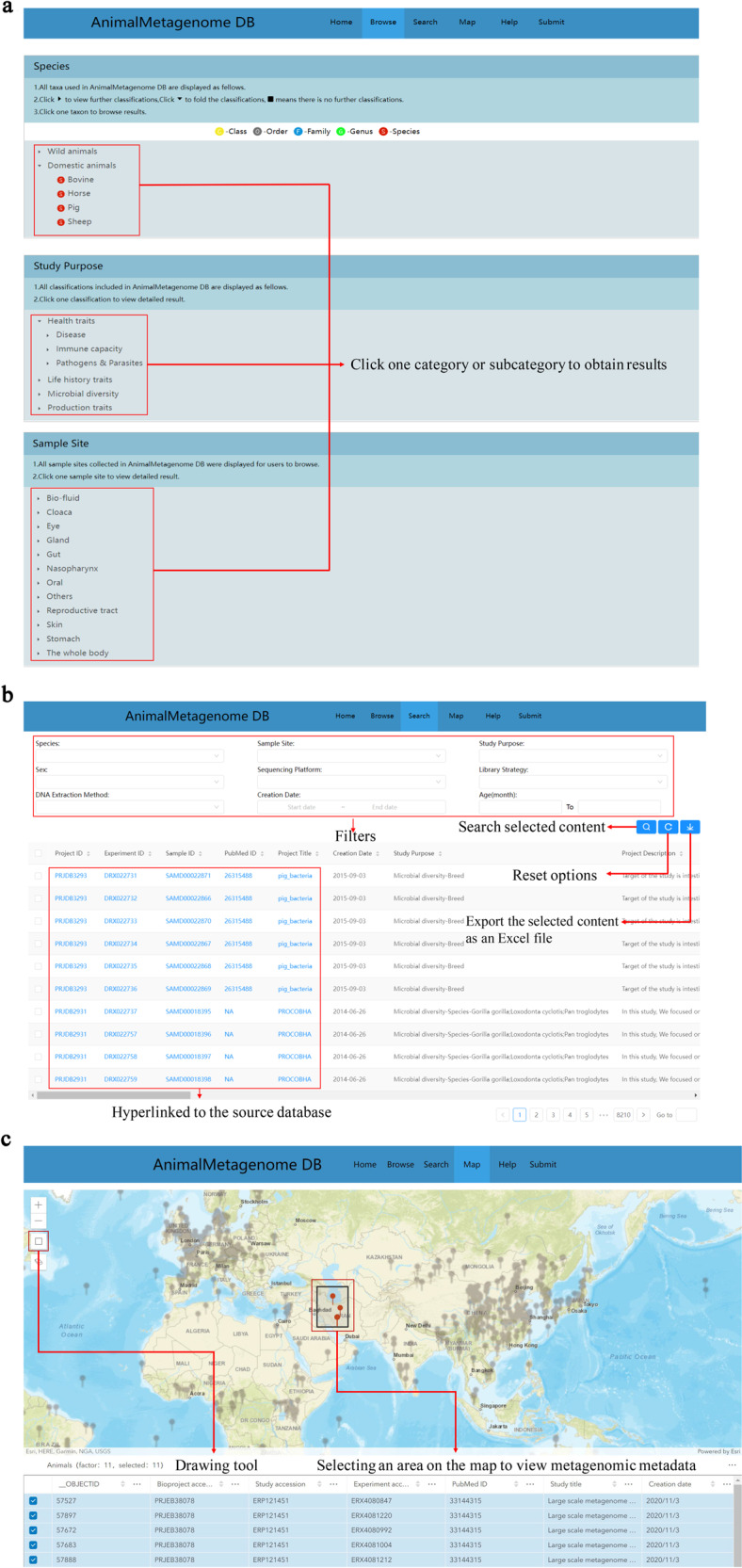
The AnimalMetagenome DB user interface. (**a**) The “Browse” page allows users to browse data. (**b**) The “Search” page allows users to select samples according to nine attributes. (**c**) The “Map” page allows users to select samples according to their geographical location on the world map.

The AnimalMetagenome DB provides a platform that can help users to browse, search and download animal metagenome metadata according to their interests. The AnimalMetagenome DB user interface is divided into three parts (Fig. 3).

“Browse”, displays the specific information of species, study purpose, and sample site. Users can choose any category or subcategory of interest to obtain results. In addition, users can further filter the dataset by selecting single or multiple filters of “Species”, “Study Purpose “, and “Sample Site” in the obtained results. After filtering, the metadata of the selected item can be downloaded as an Excel file. The queried information will be displayed below the screening box, including project ID, sample ID, experiment ID, PubMed ID, species, sample site2, study purpose, creation date, library strategy, and DNA extraction method.

“Search”, contains all the data of the current version of the database (8,2097 animal metagenomic metadata in total). The data set can be filtered according to the nine attributes “Species”, “Sample Site”, “DNA Extraction Method”, “Sequencing Platform”, “Library Strategy”, “Study Purpose”, “Creation Date”, “Sex”, and “Age”. All animal metagenomic metadata will be displayed in the search section, including samples that are not marked with geographic location. Users can filter animal metagenomic metadata by selecting single or multiple attributes of interest. After filtering, the metadata of the selected samples can be downloaded as an Excel file. The information about each sample—“Project ID”, “Project Title”, “Experiment ID”, “Sample ID” and “PubMed ID” (if present)—can be hyperlinked to the source database.

“Map”, provides a more intuitive method for reviewing data. Users can directly select samples from the world map according to the location of interest, but only select samples marked with the geographical location. This function includes the rectangular drawing tool to help user to select an area of interest on the map and then select samples. It is worth noting that a single point shown on the map may represent multiple samples. Therefore, users can only use the drawing tools to select samples in a given area. After selecting an area on the map, the selected metagenomic metadata will be displayed in the data set table below the map.

## Usage Notes

To better understand the usage of the AnimalMetagenome DB, we give an example. The microbiota is extremely important to the function and health of the gastrointestinal tract^32^. Porcine epidemic diarrhea is an enteric disease in pig caused by porcine epidemic diarrhea virus (PEDV), which is a member of the family *Coronaviridae*^33^. If we are interested in PEDV caused intestinal dysbiosis in pigs, we can use the AnimalMetagenome DB to find related studies and samples. In the “Search” interface, we can click on “Species” – “Domestic animal” – “Pig”, then select “Sample Site” – “Gut”, and finally select “Study Purpose” – “Health traits” – “Pathogens & Parasites” – “PEDV”. After selecting the filter criteria and clicking “search”, two projects and 44 samples will be shown. We can view all the metadata of the related metagenome and click “Project ID”, “Sample ID”, “Project Title”, “Experiment ID” or “PubMed ID” to open the source database. Finally, we can download the metagenomic data of the selected samples to further analyze the changes of pig intestinal microbiota affected by the porcine epidemic diarrhea virus, as well as the composition and relative abundance of gut microbiota at different taxonomic levels.

## Data Availability

The code of the AnimalMetagenome DB has been uploaded to GitHub: https://github.com/boyNextDooooor/AnimalMetagenomeDB.git.
